# Mental health care for medical staff and affiliated healthcare workers during the COVID-19 pandemic

**DOI:** 10.1177/2048872620922795

**Published:** 2020-04-01

**Authors:** Matthew Walton, Esther Murray, Michael D Christian

**Affiliations:** Northwick Park Hospital (Accident and Emergency Department), London North West University Healthcare NHS Trust, UK; Centre for Medical Education, Barts and The London School of Medicine & Dentistry, Queen Mary University of London, UK; London’s Air Ambulance, Royal London Hospital, UK

**Keywords:** Mental health, Covid-19, medical staff, critical care, physician wellbeing, PTSD, moral injury, pandemic, leadership, crisis management

## Abstract

The COVID-19 pandemic is an unprecedented challenge for society. Supporting the mental health of medical staff and affiliated healthcare workers (staff) is a critical part of the public health response. This paper details the effects on staff and addresses some of the organisational, team and individual considerations for supporting staff (pragmatically) during this pandemic. Leaders at all levels of health care organisations will find this a valuable resource.

## Introduction

COVID-19 has infected 474,204 people and there have been 21,353 deaths.^1^ These figures are rising exponentially, and the worldwide impact of this crisis is comparable with war.^2^ Medical staff and affiliated healthcare workers (staff) are under both physical and psychological pressure. For many nations this will add to an existing baseline of psychological pathology^3^ and low morale in the healthcare sector. Supporting the mental health of these individuals is a critical part of the public health response. The scope of the paper is to address some of the organisational, team and individual considerations for supporting staff (pragmatically) during this pandemic. The paper is organised as follows. In section 2 we cover how staff may experience stress during a pandemic and in sections 3 to 5 we cover what organisations, teams and individuals can do to help, before concluding in section 6.

## How staff may experience stress during a pandemic

Research^4,5^ into the psychological effects of infectious disease outbreaks such as severe acute respiratory syndrome (SARS) and pandemic flu (H1N1) shows consistent patterns of reactions and covers the experiences of staff in work, those in quarantine and those returning to work from time away sick. Challenges for staff include not only the increased workload created by such outbreaks but also fears of contagion for themselves and their families, working with new and frequently changing protocols and personal protective equipment (PPE), caring for patients who are very sick and quickly deteriorating and caring for colleagues who have also fallen ill.

In many cases resources will be stretched to the limit by an infectious disease outbreak and, as we have already seen in COVID-19, difficult decisions have to be made about who is suitable for invasive treatments such as life-support and who is not. These treatment decisions will in some cases differ from decisions that might have been made were the disease not so virulent, or the resources greater. The gravity of the situation is well understood by most healthcare professionals, less so by the public, and this can make the situation harder to adjust to.

Interpersonal issues arise from infection control measures and the use of PPE. Communication with patients is made more complicated by PPE which covers most of the face, and staff have less time to spend with each patient. Where nursing might usually take place on a 1:1 basis, in outbreaks such as COVID-19, nurses are required to treat several patients at once.^6^ This means that they are required to practice in ways which deviate from their usual standards.^7^ Family and friends may not be able to visit the patients, and staff often feel guilt that the patient has ‘died alone’.^8^ Normal routines for breaking news of death are not available, the news may have to be shared over the phone or Skype.^9^ Equally, the opportunity to view the body and collect belongings will not be available.

Many staff will contract the infectious disease, some will become very ill and some will die.^10^ Those who have been exposed or who show symptoms will be required to go into quarantine, usually away from their families. Research^4^ has shown that those staff who are quarantined experience guilt about leaving front lines understaffed, fear that they have contaminated their families and conflict about their roles^11^ as healthcare professionals and parents or carers. They also suffer from boredom, exhaustion and loneliness, especially as they usually work as part of a close-knit team. Post-quarantine they may be anxious or reluctant to return to work.

There will also be staff who will be unable to work in clinical areas where they risk the most exposure to the illness because of underlying health conditions or pregnancy. Staff in this situation, or those not posted directly on the front line for any other reason, may feel guilt.^5^ Staff are often required to work longer hours and live away from home, thus disrupting relationships and the opportunity for rest breaks and days off. Staff and their supporting families also share in the same socioeconomic disruption and restrictions as other members of the public. They may despair at the differing responses exhibited by different nations. Constant news coverage blurs the lines between home and work.^5^

Not all staff become distressed in the same way or to the same degree. Williams et al.^12^ note the ways in which people respond to emergencies and disasters fall into four main groups (Table 1).

**Table 1.** Individual reactions to disaster

Individual reactions to disaster (Williams et al.)^12^
1. **Not upset at all** (some distress but recover with the support of family members, friends or others)
2. **Proportionately distressed**, but able to function in the short and medium term (not mentally disordered)
3. **Disproportionately distressed** or distressed and dysfunctional in the short to medium term (may recover relatively quickly if given appropriate assistance as well as those who may develop mental disorders; therefore, people in this group require a thorough assessment)
4. **Mentally disordered** in the short, medium or longer term (require specialist assessment followed by timely and effective mental healthcare)
Adapted from: Williams et al.^12^

It is useful for staff to understand the variability of responses and that these will fluctuate throughout the crisis. There will also be positive responses to stressful events at work, such as post-traumatic growth.^13,14^ It is also important to note that many profound reactions of staff will still be within what is considered a ‘normal’ reaction, and in many instances will not constitute mental health pathology. Concerns have already arisen^15^ around negative psychological effects during the pandemic such as burnout, compassion fatigue, anxiety, depression, post-traumatic stress disorder (PTSD), moral injury. Not all of these will occur, nor will they necessarily last long beyond the end of the pandemic. We explore some of these in greater depth below.

### Acute stress reactions

Acute stress reactions can be significant in their presentation, but usually resolve within a couple of weeks or so. They include emotional, cognitive, physical and social reactions and will usually present in combination. It is important that staff are aware of these reactions and that it is normal to experience them, because distress arises from feeling guilt or shame about these reactions happening. See Table 2 which shows symptoms of acute stress reactions.^16,17^

**Table 2:** Indicators of acute stress reactions.^16,17^

Indicators of acute stress reactions^16,17^			
Physical	Behavioural	Emotional	Cognitive
Palpitations	Avoidance	Numbness	Poor concentration
Nausea, low appetite	Recklessness	Anxiety	Intrusive thoughts
Chest pain	Detachment	Low mood	Flashbacks
Headaches	Withdrawal	Anger, fear	Poor memory
Abdominal pains	Irritability	Mood swings	Confusion
Insomnia	Drug or alcohol use	Anhedonia	Hyper vigilance
Hyperarousal	Conflict with others	Low confidence	Rumination

### Moral injury

Moral injury is present when there is a betrayal of what is right, either by the self or by someone in legitimate authority, in a high stakes situation.^18^ While the pandemic we are currently dealing with is a sort of natural disaster, the reactions of those ‘in legitimate authority’ will be perceived by many as ‘a betrayal of what is right’. Many of those aware of this and affected by it are in healthcare and adjacent fields. It is clear that even in the most perfect of scenarios, this pandemic would have overwhelmed existing resources because of the number of patients requiring intubation and intensive care, but it is also true that more could have been done in the time available to prepare. At an individual level, clinical decisions will have to be made which contravene the morals of those making them. For example, choosing which patients will not receive life support if there are resource scarcities. These decisions will be supported by protocol, but they differ from usual practice and guidelines pre-COVID-19. Already staff report being worried about having to make these decisions, having seen the experience of their colleagues in other countries, and they are experiencing anticipatory guilt^19^ while they wait for the peak to hit in their own countries. It will be essential for leaders at all levels to remind staff that they are not making decisions alone, that there is protocol, and also to recognise that these decisions go against the grain for many. It will be necessary to offer ongoing support to all staff, whether that is a local Schwartz round^20^ type gathering or something similar, in order for these experiences to be processed for some time after the most difficult phase of this pandemic has passed.

### Post-traumatic stress disorder

Hospitals are preparing for a magnitude more deaths of patients than usual in addition to a very tangible threat of physical disability and death^21,22^ now faced by staff themselves and their families, increasing the risk of PTSD. New research suggests that PTSD in resuscitation providers at baseline is 9.6%.^23^ The risk of PTSD for front line staff in this pandemic may therefore be greater than 10%.

## What can organisations do for staff

Organisations will be keen to support their staff’s mental health and wellbeing needs during this pandemic. Resources have traditionally been put towards supporting staff once they have developed mental health pathology, for example rapid access to counselling, psychiatry and contingency for time off work. However, a shift of focus is needed from the individual to the organisation. Prevention and mitigation is far more important than cure.^24^

Organisations should immediately reflect on the challenges that staff faced at a baseline before the addition of the pandemic. Shift working, night shifts, overstaying breaks and shift ends, workload, ‘corridor medicine’ are all significant factors influencing wellbeing.^24–26^ Recognition of these factors and how the pandemic will influence them is important. The pandemic will also probably cause changes to other factors that affect wellbeing such as the structure of the organisation, roles of staff, autonomy and availability of senior support. Research has shown that individuals benefit from tangible and practical support.^5^ Organisations are able support staff in many ways (see Table 3).^15,24,25,27–30^

**Table 3.** Organisational support in a pandemic

*Organisations can support staff in a pandemic by*:
Providing food, drink and rest facilities
Ensuring staff do not exceed safe hours by encouraging reporting and monitoring of hours, and preparing reinforcements so staff can take annual leave and breaks
Focusing on dynamic workload management and clear role expectations
Proactively addressing resource inequities across the organisation
Proactively resolving housing or transport issues for staff to reduce anxiety of infecting family members and safely travelling to and from work
Regular situational updates for all staff, including realistic and frank information about risk and adverse events, e.g. report of death among colleagues or advising staff to write a will
Regular praise for staff and acknowledgement of the unprecedented and exceptional circumstances
Being visible on the ground throughout the pandemic (managers, senior staff)
Clear messaging, rationale and guidance for changing standards of practice
Encouraging a two-way dialogue and being open to suggestions and ideas from staff
Facilitating debriefs and morale building communal time
Designing rotas so that teams can stay together (despite migrating through changing shift times) throughout the pandemic
Being clear that staff safety is the number one priority
Providing adequate PPE and identifying/removing high-risk staff from frontline work to reduce anxiety for becoming infected
Providing education on the normal responses to extreme stress to reassure staff
Educating team leaders on debriefing practices and the needs of individuals
Providing formal and informal psychological support
Ensuring staff in quarantine are regularly supported and communicated with during and after their isolation
Planning specifically for supporting teams if colleagues are critically ill or deceased
Ensuring there is appropriate support for different staff grades and disciplines, e.g. doctors and nurses, as well as porters and cleaning staff
Keeping up to date with evolving guidance on supporting staff and recommendations

PPE: personal protective equipment.

### Organisational provision of psychological support

Drop-in sessions with psychologists/psychiatrists have been recommended based on evidence from previous outbreaks.^5^ The availability of support from psychologists and psychiatrists will vary from hospital to hospital and is likely to be scarce. An example of learning from a Toronto hospital in the SARS outbreak was that psychological drop-in sessions were more effective when they were offered in comfortable surroundings^5^ such as a room with sofas and music, and where senior staff also used the drop-ins. Remote psychological support such as phone, Skype etc. is indicated especially when the goal is having as few people on site and exposed to infection as possible. While peer support has its place, sometimes being able to offload to a relative stranger can be useful to staff, especially in order to acknowledge feelings they are struggling with such as fear, anger and a reluctance to come to work at all.

### Support for staff in isolation/quarantine

Issues arise due to isolation, fears of developing symptoms^4^ and fears of having brought the illness home to loved ones. Consideration should be given to assisting staff with finding alternative accommodation away from home to mitigate the fear of exposing their families. There does not seem to be any clear evidence about which staff will become very distressed when in quarantine, so it is best to offer psychosocial support to everyone. Previous research has shown that lack of adequate information and insufficiently clear guidance was a stressor for those in quarantine.^4^

Measures that can be taken to support staff in quarantine are listed in Table 4. When supporting staff, both those in quarantine/isolation at home or during ‘work isolation’ (where staff must work on infected wards, but isolate at home outside of work), it is important to remember that both psychological support related to the primary stressor (dealing with the pandemic at work) as well as support to mitigate secondary stressors (related to the basic needs of life such as childcare, grocery shopping and other basic life activities) are needed.^31^

**Table 4.** Supporting staff in quarantine

*Measures that can be taken to support staff in quarantine*:
Ensure prompt testing so that staff who are self-isolating can be confident that they are doing so appropriately
Reduce boredom: give people work to do, keep them up to date with what is going on back at the hospital – this can also help to facilitate their return to work
Encourage staff to create an exercise schedule, calming/grounding exercises, meditation
Alleviate loneliness: keep staff in touch with teams, and up to date with what is going on back at the hospital – encourage them to contact friends and family
Address guilt about leaving work ‘shorthanded’ and concerns over how they may be perceived by other staff members. Reinforce the altruism of their isolation
Offer phone/online support from a psychologist and teach stress management techniques, consider putting them in touch with a ‘survivor’ peer supporter to relate with
Arrange delivery of food, drink and medicines if required

The return to work phase should be particularly carefully handled because even brief stints away from work, such as a day off, have been shown to have quite a profound effect^4^ and staff have felt unsure about what they would be returning to. Avoidance behaviours are common in returning staff, including staff not wishing to treat highly infectious patients, absenteeism, avoiding crowded places and so on.

## What can teams do for members

### Team leaders

Although it may sound trite, the most important thing that leaders can do for their team in a crisis is be a good leader. This is certainly far harder to do than it is to write down on a piece of paper. Leadership during a crisis is always a challenge; however, leading during the COVID-19 situation is even more difficult given that leaders themselves are ‘living’ in the crisis and are equally impacted by it as much as those who they are leading. Despite the tragedy of the current situation it is important to recognise that crises are also times when new leaders are often ‘born’ and many existing leaders excel. Although there are many negative aspects of the current situation, teams can grow stronger, individuals can develop, relationships can grow deeper as a result of this crisis. The impact of this pandemic and how leaders respond during it will shape the future relationship of teams and culture of organisations for years to come. Table 5 provides a list of both strategies and tactics leaders can draw on to support their teams through this crisis. We highlight three key considerations below.

**Table 5.** Crisis leadership strategies

*Crisis leadership strategies*	
Acknowledge the gravity of the situation	Create talking points for managers/the team
Actions speak louder than words	Debrief teams after critical incidents
Assume and demonstrate responsibility	Empower and inspire others
Balance expertise and intuition to act decisively in uncertainty	Encourage them to be their own leaders
Be connected	Explain ‘meaning-making’
Be decisive and confident	Forge a path together
Be flexible	Know others’ strengths and weaknesses
Be human	Listen to your team
Be humble	Look at the big picture
Be present	Model good behaviours and practices, including good self-care
Build on the strengths of others	Motivate others
Build on your strengths	Normalise reactions to stress
Build resilience to cope with prolonged high-stress situations	Prioritise what is truly important
Choose a positive future	Remain calm
Clearly outline support resources	Stay organised
Communicate, communicate, communicate	Set boundaries

Communication: as is true in almost all cases, it is often impossible for leaders to communicate too much. The challenge in this pandemic is what to communicate. A key principle behind communication in this setting is to be open and honest: say what you know are facts, say what you don’t know but what you are going to do to find out. While maintaining honesty, it is still important to remain calm, motivate your team and help them look beyond the current crisis to positive days ahead. Finally, remember communication must go in both directions. Ensure you take time to listen and create multiple different avenues for your team to ask questions, provide feedback and share their feelings or concerns.

Empowerment: there is no time more important than the present for leaders to encourage individuals to be their own leaders. Teams always needs leaders but with this pandemic, which is likely to last for months, expect that with the demands of the pandemic (physical and psychological) on leaders as well as a predicted 20% absenteeism due to illness or quarantine, it will undoubtedly be necessary for the baton of leadership to be passed between people during this marathon. By encouraging people to lead themselves people will be better prepared to be able to step up when needed.

Humanity and humility: key to supporting a team during this pandemic is understanding the humanity of the situation. Leaders must recognise both the strengths and limits of themselves and those of their team. Acknowledge and normalise feelings of fear and anger, which anyone might feel in this situation, and help people make meaning of their experience. It is essential leaders themselves model good practice and behaviours for coping, including seeking help and self-care. Finally, it is not uncommon to question one’s capacity to lead in situations such as this. It is important, while still being humble, to be confident and assume and demonstrate responsibility. If you are a leader and have chosen to read this article, you have demonstrated you already have what is required to be a successful leader in this crisis.

### Colleagues

The importance of peer support should not be underestimated. Peer groups have shared experiences, which means they are able to communicate in a kind of shorthand. There is no need for members of the peer group to worry about breaking taboos because their social rules are already established, and they can speak to one another more freely than they may to friends and family. When peer support is formalised, for example by training welfare ambassadors or other peer supporters, those coordinating psychosocial support in each department should offer debrief/supervision sessions for peer supporters.

Colleagues and friends at work that can support each other will be an important part of maintaining good wellbeing and camaraderie^4^ during the pandemic. See Table 6 for a list of ways colleagues can support each other.

**Table 6.** Ways colleagues can support each other

As a colleague you can support your co-workers by:
Spotting signs of concern in them (nightmares, difficulty sleeping, unable to stop worrying, jumpy, easily irritable, medically unexplained symptoms appearing, flashbacks of stressful events)
Offering them the opportunity to talk (do not force them to do so, but be available to listen, laugh or cry with them)
Signposting them to supportive resources
Being kind, consistent and reassuring
Encouraging them to maintain good self-care
Helping them explore the cause of their distress, and if you can help them address it, or if you need to escalate concerns

## What can individuals do for themselves

In this time of extreme anxiety and uncertainty, it is worth remembering what we can do for ourselves. Many of the habits of mind that have stood us in good stead up to now will still hold true throughout this pandemic. It will be useful to be able to acknowledge the gravity of the situation, noticing what is in your control and what is not. Psychological reactions to the acute and prolonged stress of the pandemic will be common and you are likely to experience these – it does not mean they will last long term or that you are ‘weak’. Remember that it is the virus that kills people, not the staff.

This pandemic is a marathon and not a sprint – take your breaks, try to keep leave and take time to ‘reset’ yourself. It is important, although not always easy, to allow yourself to rest and reset, but here are some practical things you can try. Starting small can be easier. Remember to eat, drink, sleep and exercise first, and try to focus on positives and be thankful for the good things in your life, for example family and friends.

Keep in regular contact with family and friends by phone or Skype and let yourself talk about your feelings and share your experiences with others. It may feel much more appropriate not to talk about work, and this is also fine. Take time to rationalise the risks of infection to yourself and your family so you feel comfortable going to work. Know who forms your peer support network and where to access external support should you require it. Allow yourself to be proud of your important role in society and the work that you are doing to help others.

## Conclusion

The COVID-19 pandemic is unprecedented. Its impact will likely be imprinted on each individual involved. Widespread stressors will emerge or become exacerbated. Many staff will be negatively psychologically affected.^32^ There are, however, opportunities at every level to make a difference to the mental health support of staff, and to identify and encourage opportunities to find growth and meaning in this situation. Our society should now regard these individuals as ‘gold dust’ and it is our duty to provide the support they deserve.

### Recommended links

https://www.supporttheworkers.org/.

Williams et al. checklists – from paper on Supporting staff after disasters: https://www.apothecaries.org/wp-content/uploads/2019/02/OP94.pdf.
